# Cribbing, and Its Operative Treatment

**Published:** 1899-01

**Authors:** S. J. J. Harger

**Affiliations:** Professor of Veterinary Anatomy and Zootechnics, University of Pennsylvania


### CRIBBING, AND ITS OPERATIVE TREATMENT.[1]

By S. J. J. Harger, V.M.D.,
PROFESSOR OF VETERINARY ANATOMY AND ZOOTECHNICS, UNIVERSITY OF PENNSYLVANIA.

This abnormal act, too well known to be defined here, is seen in all races and breeds of horses.

Among its causes have been mentioned idleness, prolonged stabulation, and tying of young horses in the stable, temperament, hunger, imitation, etc. Evidence as to its being hereditary is not conclusive.

The theory of its being a neurosis, with muscular spasm, or an eructation of gas from the stomach is now abandoned. It is possible that the act may for the first time be accomplished accidentally by the animal licking and biting the manger and grasping the latter with the teeth more firmly from day to day until he employs sufficient effort to complete the act. A frequent repetition of this practice may in time lead to a deviation of the normal function of the nerve-centres controlling the muscular contraction, and thus create a constant desire to crib, which might be termed an

[1] Digest from clinical lecture, October, 1898.

acquired neurosis, so obstinate to treatment because of the lack of will and moral power in the lower animals.

The *mechanism* of cribbing consists: 1. A fixation of the head on some object, generally with the teeth. 2. Muscular contraction accompanied by expansion of the chest, cervical muscles, anterior straights, longus colli, sterno-maxillaris, rhomboideus, trapezius, sterno, hyo, thyroideus, angularis, the pectorals, intercostals, and diaphragm. 3. Influx of air into the pharynx, larynx, and bronchi, accompanied by the characteristic sound. The air may not pass beyond the entrance into the œsophagus, only to be regurgitated immediately afterward. Dieckerhoff (*Koppen des Pferdes*, 1897) estimates that only in 5 per cent. of cribbers does the air enter the stomach. Even in such a case the act is not one of *deglutition*, the phenomena in the two being quite different; but it may explain the differences seen in the general condition of cribbing horses as well as the digestive troubles. The muscles named above are always hypertrophied.

This description does not include the phenomenon ordinarily called "windsucking," which, in at least a few observations that I have seen, appeared to be a swallowing of air in its true sense; and in these cases a few minutes suffice sometimes to produce considerable bloating.

The *symptoms*, such as the wear of the teeth, which requires from three to four months to become evident, the general condition, etc., are well known.

The *prognosis* varies. More favorable when the air does not enter the stomach, which causes the digestive and constitutional symptoms.

*Treatment.* The various mechanical contrivances as to the halter and bits have been found ineffectual. The neck-strap, composed of leather, hair, or wire, by compressing the cervical muscles and preventing their contraction, is a palliative means, but does not cure the trouble. I intend to speak more especially of the operative treatment.

Various operations have been suggested: 1. Section of the tendon of the sterno-maxillaris muscles. The result was only temporary, as the cut ends of the tendon subsequently united (Goubaux, Hering, Bossi). 2. Section of the same muscle below where it receives the spinal accessory nerve. 3. Resection of the subscapulo-hyoid muscle. 4. Resection of the sterno, hyo, thyroid muscles. 5. Neurectomy of the spinal accessory nerve.

In two of my cases the fourth operation was performed, while in the other two subjects the fourth and fifth operations were done in the same animals.

1. *Resection of the sterno-, hyo-, thyroid muscles.* The point of election is the superior part of the anterior border of the neck, where tracheotomy is usually performed—that is, above the point where the sterno-maxillaris muscles separate to pass across the side of the trachea, and where the latter is separated from the skin only by the cuticularis colli muscle and the muscles to be resected. The skin is shaved and treated for twenty-four hours with the usual antiseptic practice, cleansed with soap and water, then with a bichloride solution and a pad of oakum soaked in bichloride solution applied with a bandage. A median incision, about one and one-half inches long, is made through the skin and the cuticularis muscle; the lips of the wound are then separated with blunt retractors. The muscles are freed from the surrounding connective tissue with two pairs of dissecting forceps, and a grooved director is passed behind them. They are then separated from the trachea, and after securing them with a ligature they are cut as far upward toward the larynx as possible; the lower section is made so as to remove about two inches of muscle. As the distal stump will retract and form a subcutaneous pocket below, a rubber drainage-tube, directed downward, is inserted into the lower commissure of the incision. Three sutures suffice. A bichloride oakum dressing is applied and the parts treated like any other wound. Cicatrization may follow by primary union.

These muscles are selected because it is supposed that by their contraction they depress the laryngo-pharyngeal apparatus in order to open the entrance into the œsophagus to admit the air.

2. *Neurectomy of the spinal accessory nerve (twelfth cranial pair).* A branch of this nerve constitutes the motor supply of the sterno-maxillaris muscle, entering that muscle at its anterior extremity. This muscle is in close contact along its superior border (neck horizontal) with the jugular vein, which must not be injured. By compressing the jugular vein its distended glosso-facial branch can be seen crossing obliquely the tendon of this muscle a short distance below its attachment to the angle of the lower jaw. The incision through the skin and cuticular muscle, commencing about a half an inch below the glosso-facial vein, is made opposite to and parallel with the superior border of the muscle and about two inches in length, leaving the jugular vein above. With the fingers or a tenaculum the inner face of the muscle is everted toward the incision. After pushing the connective tissue to one side the nerve branch will at once appear in view. The nerve is then secured with a ligature and a portion resected. The muscle, which, during the handling of the nerve, contracted spasmodically, is now perfectly relaxed. The previous as well as the subsequent treatment is the same as in the other operation; no drainage-tube is inserted. The object of resecting this nerve is to prevent the animal from firmly fixing his head on a foreign object. The resulting scars are insignificant. When returned to his stall the animal usually makes a few ineffectual attempts to crib, which are soon abandoned.

A few fragmentary cases, but with only incomplete details, are recorded, hence the following notes with conclusions from cases at the veterinary hospital.

Case I.—Bay gelding, cribbing incessantly; condition poor. Operation in June, 1898. Resected the sterno-hyoid and sterno-thyroid muscles. After the operation the act was sometimes accomplished successfully; at others unsuccessfully. At present writing the animal still cribs at times, but in this respect, as well as in his general condition, is very much improved.

Case II.—Roan gelding. Cribbing not as aggravated as in previous case. Same operation. Cribs only about half as often as before, and does not show the same desire.

Case III.—Black mare. Cribbing incessantly on any object, even the halter-strap. Resected the muscles as in Case I. Also the spinal accessory nerve branch supplying both sterno-maxillaris muscles. The animal tried to crib once immediately after the operation, but has not been observed attempting to do so since.

Case IV.—Bay gelding, condition poor. Cribbed like Case. III.; performed the same operation. Has not been known to crib since.

From these four cases it would seem to me that, while resection of the muscle alone much lessens the frequency of cribbing, it does not effect a cure. On the other hand, the indications are that the double operation, as practised in Cases III. and IV., seems to remove the trouble entirely. Without drawing any positive conclusions as regards the curative value of these operations, the facts of the last two cases seem to encourage us to anticipate favorable results in future cases. Lapse of time and more cases will determine the question. We shall also experiment with the so-called "windsucking," in which the phenomena are somewhat different from those seen in the cases here cited.

